# The Duration of Cataract Surgery and the Change in Postoperative Dry Eye Disease Parameters: A Retrospective Cohort Study

**DOI:** 10.3390/diagnostics15030330

**Published:** 2025-01-30

**Authors:** Chia-Yi Lee, Shun-Fa Yang, Yun-Chen Chen, Chao-Kai Chang

**Affiliations:** 1 Institute of Medicine, Chung Shan Medical University, Taichung 40201, Taiwan; 2 Nobel Eye Institute, Taipei 10041, Taiwan; 3 Department of Ophthalmology, Jen-Ai Hospital Dali Branch, Taichung 41265, Taiwan; 4 Department of Medical Research, Chung Shan Medical University Hospital, Taichung 40201, Taiwan; 5 Chaomuhean Clinic, Taipei 10689, Taiwan; 6 Department of Optometry, Yuanpei University of Medical Technology, Hsinchu 30015, Taiwan

**Keywords:** dry eye disease, tear break-up time, ocular surface staining, cataract surgery, surgical time

## Abstract

**Objective**: The aim of this study was to evaluate the correlation between the duration of cataract surgery and the postoperative dry eye disease (DED) parameters. **Methods**: A retrospective cohort study was conducted on individuals who received cataract surgery at our institution. In total, 72 and 36 eyes were split into the short and long surgery groups, respectively. The main outcomes were the postoperative tear break-up time (TBUT), the Schirmer II test result, the ocular surface staining score, the DED symptoms, and the presence of meibomian gland dysfunction (MGD). A generalized linear model was employed to compare the changes in the DED parameters between the two groups. **Results**: The uncorrected visual acuity (UDVA) was significantly better in the short surgery group compared to the long surgery group (*p* = 0.031). The latter group also demonstrated a significantly shorter TBUT and a higher ocular surface stain score compared to those of the short surgery group (both *p* < 0.05). The change in the TBUT and ocular surface stain score were significantly higher in the long surgery group than the short surgery group (both *p* < 0.05). No DED parameters were significantly associated with the postoperative UDVA in the short surgery group (all *p* > 0.05). However, the TBUT and the ocular surface stain score correlated with worse postoperative UDVA in the long surgery group (both *p* < 0.05). **Conclusions**: Operating on a cataract for more than 20 min correlates with a long TBUT and a poor ocular surface staining score, which could influence the postoperative UDVA.

## 1. Introduction

A cataract is an opaque part in a crystalline lens which can disturb visual acuity to a prominent degree [1]. About 92 percent of individuals diagnosed with senile cataracts are 80 years old [2]. To manage the reduced visual acuity resulting from cataracts, cataract surgery is the only effective approach, and more than one million people undergo this treatment every year [2,3]. The postoperative visual outcome of cataract surgery is generally acceptable. The introduction of a presbyopia-correcting intraocular lens (IOL) can additionally improve the near visual acuity [4,5].

Although the majority of people who receive cataract surgery gain improved visual acuity, certain complications can still occur [3]. Postoperative infectious endophthalmitis may be the most dreaded complication of cataract surgery, causing permanent visual loss; additionally, eyeball removal could be advocated [6]. In addition, posterior capsular rupture can also retard the speed of visual recovery after cataract surgery, so sulcus IOL implantation or even IOL fixation will be needed [7]. Postoperative ocular hypertension and subsequent glaucoma are other complications that affect a person’s postoperative vision [8]. Additionally, persistent corneal edema and bullous keratopathy after cataract surgery can reduce their vision significantly [9].

Postoperative dry eye disease (DED) is a complication that affects about 37 percent of individuals who have received cataract surgery [10]. Postoperative DED may result from damage to the corneal surface and the thermal effect of the phacoemulsification technique [11]. The known risk factors of DED are similar with or without the receipt of cataract surgery, which include previous DED history, old age, the female sex, ocular surface inflammation, and having undergone refractive surgery [12,13,14]. Still, it is unknown whether the exact duration of cataract surgery affects the incidence of postoperative DED. Since the persistent exposure of the cornea can cause DED [11], prolonging the duration of cataract surgery may result in postoperative DED, which will need additional evaluation.

Thus, we aim to evaluate the effect of cataract surgery duration on the change in postoperative DED parameters. Other risk factors for DED development were also analyzed in this study.

## 2. Materials and Methods

### 2.1. Participant Selection

This retrospective cohort study was conducted at the Nobel Eye Institute, which represents several clinical groups with more than 15 branches throughout the Taiwan area. Individuals were included in this study if they met the following criteria: (1) they had been diagnosed with senile cataracts in any branch of the Nobel Eye Institute, (2) they had received cataract surgery at any branch of the Nobel Eye Institute, (3) they had been followed up with at any branch of the Nobel Eye Institute for at least three months, and (4) they were older than 55 years. In addition, the following exclusion criteria were applied to exclude those individuals with an impaired ocular surface: (1) the presence of a corneal ulcer before cataract surgery, (2) the presence of recurrent corneal erosion before cataract surgery, (3) the presence of corneal neovascularization before cataract surgery, (4) the presence of any form of corneal endothelial dystrophy before cataract surgery, (5) the presence of extremely severe DED that accompanies diffuse puntate keratitis before cataract surgery, and (6) the presence of a chemical burn before cataract surgery. Then, surgical records were obtained, and the duration of each cataract surgery was collected. We divided the individuals into short and long surgery groups. A surgical time of 20 min served as the threshold/watershed. We set this threshold/watershed because a surgical time of within 20 min was regarded as acceptable by all the authors. A person who had undergone surgery on one eye for more than 20 min was matched with another person whose both eyes had been operated on for less than 20 min within three months of initial surgery. Of note, only the first eye of each individual that underwent cataract surgery was included in this study. Finally, 72 and 36 eyes were included in the short and long surgery groups, respectively.

### 2.2. Cataract Surgery

All the cataract surgeries in this study were conducted by one cataract specialist (C.-Y.L.), and one phacoemulsification device was utilized (Quatera, Carl Zeiss, Göschwitzer Str., Jena, Germany). The IOL power was calculated via the online Barrett formula. The main incision was made in the superior portion, and then the ophthalmic viscoelastic device was released into the anterior chamber. After continuous curvilinear capsulorhexis, hydrodissection via the side-port was performed. The phaco-chop technique was performed on the nucleus, and then the cortex material was handled using an infusion/aspiration tip. An IOL was then implanted into the capsular bag, and the remaining ophthalmic viscoelastic device was cleaned. The hydroseal approach was adopted to close the corneal incision, and finally, moxifloxacin eyedrops and tobradex ointment were instilled. After the cataract surgery, levofloxacin and prednisolone eyedrops and tobradex ointment were used for one week and then changed to combined dexamethasone and neomycin eyedrops for another one week. Sulfamethoxazole and fluorometholone agents were applied for approximately three weeks.

### 2.3. Dry Eye Evaluation

All the individuals in this study underwent the same preoperative and postoperative exams. Preceding ophthalmic disorders and surgeries were recorded if they occurred. The preoperative exams examined the uncorrected visual acuity (UDVA); cycloplegia refraction, using an autorefractor (KR-8900, Topcon, Itabashi-ku, Tokyo, Japan); and intraocular pressure (IOP), via pneumatic tonometry (NT-530, NIDEK, Gamagori, Aichi, Japan). Cycloplegia refraction is exhibited as a spherical equivalent (SE) in this study, which means the sphere power plus half of the cylinder power, and presented as diopter (D). Regarding the DED-related indexes, a tear break-up time (TBUT) assessment, a Schirmer II test, and fluorescein ocular surface staining were performed on all the individuals. The ocular surface stain score was based on the Oxford Scheme. The presence of meibomian gland dysfunction (MGD) was also decided by inspection. For the DED symptoms, dryness, a burning sensation, grittyness, soreness, fatigue, itching, a foreign body sensation, redness, photophobia, and discharge were recorded using the medical documents. The postoperative examinations also included the UDVA, IOP, and manifest refraction using the exact same devices. Moreover, ocular surface staining, the TBUT, the Schirmer II test score, and the presence of MGD were determined after cataract surgery. The number of DED-related symptoms was also recorded postoperatively if they existed. The examinations before and one month after cataract surgery were analyzed in this study.

### 2.4. Statistical Analysis

SPSS version 20.0 (SPSS Inc., Chicago, IL, USA) was used for all the statistical analyses in this study. The statistical power of this study was 0.85, with a 0.05 alpha value and a medium effect size, which was yielded using G∗power version 3.1.9.2 (Heinrich Heine Universität at Düsseldorf, Germany). A Shapiro–Wilk test was run to evaluate the normality of all the data, and the results exhibited normal distributions (all *p* > 0.05). A descriptive analysis was conducted for the baseline characteristics of the short and long surgery groups. Independent and Chi-square tests were performed to compare the preoperative indexes and postoperative outcomes between the two groups. After this, a generalized linear model was used to investigate the alterations in the TBUT, the DED-related symptoms, the ocular surface stain score, the presence of MGD, and the Schirmer II test results after the cataract surgery between the two groups. The adjusted odds ratio (aOR) and related 95% confidence interval (CI) were decided upon after adjustments for age and sex in the multivariable analysis. In the next step, we separately evaluated the correlation between the worse postoperative UDVA results and the DED-associated parameters the in short and long surgery groups via the generalized linear model again. Moreover, the participants in both groups were stratified according to sex. Then, the generalized linear model was used to evaluate the postoperative DED-related parameters between the male and female populations in both the short and long surgery groups. The statistical significance was described as *p* < 0.05, and a *p* value of lower than 0.001 was described as *p* < 0.001.

## 3. Results

The basic characteristics of the two groups are presented in Table 1. The mean ages were 65.36 ± 7.46 and 64.98 ± 7.59 years old in the short and long surgery groups, respectively, without a significant difference (*p* = 0.804). Additionally, the sex distribution, the rate of previous ocular disease occurrence, and the state of having had refractive surgery did not illustrate significant differences between the two groups (all *p* > 0.05). Regarding the ophthalmic parameters, all the preoperative parameters demonstrated similar values between the short and long surgery groups, respectively (all *p* > 0.05) (Table 1).

After the one-month follow-up, the UDVA was significantly better in the short surgery group compared to that of the long surgery group (0.11 ± 0.06 versus 0.15 ± 0.08, *p* = 0.031), while the SEs were similar between them (*p* = 0.713). Regarding the postoperative DED status, the long surgery group demonstrated a significantly shorter TBUT (7.33 ± 2.68 versus 5.49 ± 2.74, *p* = 0.001) and a higher ocular surface stain score (1.84 ± 1.25 versus 2.78 ± 1.40, *p* < 0.001) compared to those of the short surgery group (Table 2). On the other hand, the values of the Schirmer II test, DED symptoms, and the presence of MGD did not illustrate a significant difference between the two groups (all *p* > 0.05) (Table 2). In the multivariable analysis, the changes in the TBUT (aOR: 1.326; 95% CI: 1.017–1.545; *p* = 0.025) and the ocular surface stain score (aOR: 1.388; 95% CI: 1.103–1.695; *p* = 0.011) were significantly bigger in the long surgery group than the other group (Table 3).

No DED parameters were significantly associated with the postoperative UDVA in the short surgery group (all *p* > 0.05). Nevertheless, the TBUT (aOR: 1.432; 95% CI: 1.096–1.728; *p* = 0.017) and the ocular surface stain score (aOR: 1.583; 95% CI: 1.207–1.840; *p* = 0.006) correlated with worse postoperative UDVA in the long surgery group (Table 4). The analyses showed that the female population showed a shorter postoperative TBUT and more ocular surface staining than the male population did in both the short and long surgery groups (all *p* < 0.05). In addition, the female population revealed more DED symptoms than the male population did in the long surgery group (*p* = 0.008).

## 4. Discussion

In this study, the postoperative UDVA, TBUT, and ocular surface staining results were significantly better in the short surgery group. Moreover, the duration of the TBUT and the ocular surface staining score changes were significantly higher in the long surgery group than they were in the other group. In addition, a shorter TBUT and more ocular surface staining were associated with worse postoperative UDVA in the long surgery group.

DED can be induced by several factors, as mentioned in previous studies [15]. From a pathological perspective, inflammation is the crucial component for the induction of the DED process, which features tear film instability and the release of cytokines [16,17]. In addition to inflammation, elevated oxidative stress and ocular surface damage could also trigger the development of DED [15,18]. From a clinical perspective, the usage of a visual display terminal device is an important factor for DED development in modern society. More than 60 percent of visual display terminal users have DED [19]. Less blinking during the use of a computer, tablet, or cell phone can damage the ocular surface and contribute to DED development [20]. Moreover, lagophthalmos, which means incomplete eyelid closure, is also associated with the development of DED; superficial keratitis is not uncommon in such a situation [15,21]. Keratorefractive surgery is another risk factor for DED development, in which more than half of individuals who undergo this procedure suffer from DED a few months after [22,23]. DED used to be more severe for those who received laser in situ keratomileusis, and the large corneal incision may be the main reason why [24,25,26]. On the other hand, cataract surgery could lead to the occurrence of DED, although it is less common after this procedure than post-keratorefractive surgery-induced DED is [10,13]. The creation of a corneal incision, the thermal effect of the phacoemulsification procedure, and the exposure of the corneal surface to air may cause post-cataract surgery-induced DED [14]. Because cataract surgery itself can increase the risk of DED, and corneal exposure can encourage DED development [10,11], cataract surgery of a longer duration may be associated with DED. This hypothesis was supported by the findings of this study, at least to some degree.

In this study, extended cataract surgery correlates with a long TBUT, a poor ocular surface stain score, and the increased deterioration of these two parameters postoperatively. In the previous study discussing post-cataract surgery-induced DED, a higher risk of postoperative DED was found in the female patients [27], and autoimmune disease also relates to a higher degree of post-cataract surgery-induced DED [14]. Concerning the influence of surgical time on post-cataract surgery-induced DED, a previous study demonstrated a positive correlation between microscope exposure time and post-cataract surgery-induced DED [28], while another study illustrated that the microscopic light exposure interval during cataract surgery is not significantly associated with the development of postoperative DED [29]. Moreover, a review article stated that longer ocular exposure during cataract surgery may be related to damage to the microvilli structure of the ocular surface and could increase the risk of postoperative DED [30]. Although the effect of surgical time on post-cataract surgery-induced DED has not been confirmed, a few articles have evaluated the influence of surgery time with the absolute value on midterm post-cataract surgery-induced DED. However, the effect of DED parameters on visual acuity in patients with different surgical durations has not been revealed. To our knowledge, this study may preliminarily demonstrate the correlation between the exact duration of cataract surgery and changes in the postoperative DED parameters. Moreover, all the preoperative DED-related parameters were statistically insignificant between the short and long surgery groups; thus, the DED status is homogenous. Furthermore, in the multivariable analysis, we adjusted for age and sex, which are well-known risk factors for DED [12], while evaluating the alteration in the TBUT and the ocular surface staining score after the cataract surgery between the two groups. As a consequence, extended cataract surgery may be an independent indicator for the development of postoperative DED. Exposing the cornea for longer during cataract surgery may be the main reason for the higher risk of DED in the long surgery group, which is similar to previous findings [11]. On the other hand, taking a long time to perform cataract surgery may be due to the advanced cataract grade and the associated ultrasound duration [31]. Still, the cataract grades between the two groups were statistically insignificant; thus, the thermal effect due to the advanced cataract status may not be the major etiology of postoperative DED in the long surgery group. Moreover, the longer TBUT and larger ocular surface staining alterations in the long surgery group indicate that surgical duration relates to postoperative DED, even in patients with the same preoperative status. Thus, a reduction in the surgical time could indeed have benefited the patients who were scheduled for cataract surgery in this study, which corresponds with the previous studies [28,30]. On the other hand, the female population showed a worse DED status compared that of their male counterpart in both the groups, which further confirms that being female is a risk factor for DED development, as reported in a previous study [12].

A shorter TBUT and a higher ocular surface stain score were correlated with worse postoperative UDVA in the long surgery group. No research has represented this relationship before. A lower TBUT indicates an unstable tear film and is a crucial parameter for DED assessment [17]. Prominent DED can influence both visual acuity and quality, as mentioned in a previous study [32]; thus, individuals with a shorter TBUT may have reduced vision, especially postoperatively. In addition, ocular surface staining reflects how intact it is; the highest grade of ocular surface staining indicates diffuse puntate keratitis and corneal damage [33]. In such a condition, visual acuity could also be diminished. Accordingly, we speculate that the prominent TBUT reduction and ocular surface staining in the long surgery group caused the decrease in the postoperative UDVA. On the other hand, the postoperative UDVA in the short surgery group was not affected by any of the DED-related parameters in this study. Perhaps the degrees of the TBUT decrement and ocular surface staining in the short surgery group were only modest compared to those of the other group. Thus, they did not cause significant ocular surface damage and subsequent visual acuity deterioration. These results further highlight the importance of short cataract surgery. The Schirmer II test results, the DED symptoms, and MGD did not affect the visual acuity of either the short or long surgery groups. This may be due to the mild-to-moderate changes in these parameters in both the populations, which did not impact the ocular surface to a great extent.

Concerning the postoperative outcomes of cataract surgery in this study, the mean postoperative UDVA scores were 0.11 and 0.15 in the short and long surgery groups, respectively. In a previous study, the mean postoperative UDVA value was 0.07, while another study demonstrated a postoperative UDVA score of 0.08 [34,35]. Our results for the short surgery group are compatible with previous data [34,35]. The postoperative SE values were −0.43 D and −0.48 D in the short and long surgery groups, respectively. In the previous articles, the mean postoperative SE scores ranged from about −0.10 D to −0.20 D, and the refraction accuracy of this study was similar to that of earlier publications [36,37]. Six individuals experienced postoperative corneal edema, which persisted for about three to five days. No other prominent intraoperative or postoperative complications were observed in our study population. In short, the postoperative outcomes at our institution are acceptable.

There are certain limitations in this study. Firstly, the retrospective design of our study may have reduced the integrity of our results and increased the heterogeneity of our study population. Secondly, the relatively small sample size, with only 108 eyes enrolled in this study (not all the patients routinely received DED-related exams after cataract surgery), may contribute to the statistical bias of our analyses despite the statistical power of the study population not being very low. Moreover, the follow-up period of this study was too short, so only one-month-postoperative data were obtained, which represents an interval with significant DED variation. The DED status may have changed after this point in time. Finally, we did not perform various DED examinations, including the tear meniscus height, lipid thickness, or blinking rate assessments, which would decrease the accuracy of our DED approach.

## 5. Conclusions

In conclusion, undergoing cataract surgery for more than 20 min correlates with a longer TBUT and more ocular surface staining. Furthermore, these outcomes are associated with the postoperative UDVA in patients who underwent surgery for a long time. Consequently, cataract surgery should be reasonably shortened, especially for those with a prior DED diagnosis. Future large-scale prospective studies to investigate whether the surgical duration has a long-term effect on post-cataract surgery-induced DED are essential.

## Figures and Tables

**Table 1:** The basic characteristics of the two groups.

Feature	Short Surgery Group (N: 72)	Long Surgery Group (N: 36)	*p*
Age (years, mean ± SD)	65.36 ± 7.46	64.98 ± 7.59	0.804
Sex (male: female)	43:29	19:17	0.539
Ocular disease			0.364
Retinal disease	2	1	
Glaucoma	0	1	
Refractive surgery	1	0	0.999
UDVA (LogMAR)	0.41 ± 0.13	0.37 ± 0.16	0.166
SE (D)	−2.89 ± 1.76	−3.02 ± 1.53	0.707
Cataract grade			0.715
Grade 1–2	7	2	
Grade 3–4	65	34	
TBUT	11.28 ± 3.67	10.89 ± 4.02	0.615
Schirmer II test	15.45 ± 6.98	16.37 ± 6.54	0.511
Ocular surface stain score	1.35 ± 1.14	1.38 ± 1.23	0.900
Number of DED symptoms	2.68 ± 1.84	2.57 ± 1.66	0.763
MGD presence	18	8	0.815

D: diopter; DED: dry eye disease; MGD: meibomian gland dysfunction; N: number; SD: standard deviation; SE: spherical equivalent; TBUT: tear break-up time; UDVA: uncorrected distance visual acuity.

**Table 2:** Ophthalmic parameters of two groups after cataract surgery.

Outcome	Short Surgery Group (N: 72)	Long Surgery Group (N: 36)	*p*
UDVA	0.11 ± 0.06	0.15 ± 0.08	0.031 *
SE	−0.43 ± 0.64	−0.48 ± 0.71	0.713
TBUT	7.33 ± 2.68	5.49 ± 2.74	0.001 *
Schirmer II test	12.52 ± 6.31	11.09 ± 5.84	0.258
Ocular surface stain score	1.84 ± 1.25	2.78 ± 1.40	<0.001 *
Number of DED syndromes	2.96 ± 1.46	3.55 ± 1.63	0.060
MGD presence	18	9	0.999

DED: dry eye disease; MGD: meibomian gland dysfunction; N: number; SE: spherical equivalent; UDVA: uncorrected distance visual acuity. * Denotes significant difference between groups.

**Table 3:** Degree of dry eye parameter change between short and long surgery groups.

Outcome (Reference: Short Surgery Group)	aOR (95% CI)	*p*
TBUT	0.786 (0.617–0.945)	0.025 *
Schirmer II test	0.867 (0.723–1.186)	0.223
Ocular surface stain	1.348 (1.103–1.695)	0.011 *
DED symptoms	1.164 (0.925–1.383)	0.142
MGD presence	1.003 (0.982–1.075)	0.816

aOR: adjusted odds ratio; CI: confidence interval; DED: dry eye disease; MGD: meibomian gland dysfunction; TBUT: tear break-up time. * Denotes significant difference between groups.

**Table 4:** The association between worse postoperative visual acuity and the dry eye parameters.

Outcome	aOR (95% CI)	*p*
Short surgery group		
TBUT	1.201 (0.944–1.368)	0.538
Schirmer II test	0.972 (0.863–1.156)	0.667
Ocular surface stain	1.007 (0.935–1.221)	0.726
DED symptoms	1.267 (0.797–1.643)	0.314
MGD presence	0.988 (0.961–1.045)	0.825
Long surgery group		
TBUT	1.432 (1.096–1.728)	0.017 *
Schirmer II test	1.283 (0.937–1.529)	0.226
Ocular surface stain	1.583 (1.207–1.840)	0.006 *
DED symptoms	1.397 (0.991–1.671)	0.094
MGD presence	1.058 (0.929–1.163)	0.688

aOR: adjusted odds ratio; CI: confidence interval; DED: dry eye disease; MGD: meibomian gland dysfunction; TBUT: tear break-up time. * Denotes significant difference between groups.

## Data Availability

The data used in this study are available from the corresponding author upon reasonable request.

## Abbreviations

The following abbreviations are used in this manuscript:

aOR	adjusted odds ratio
CI	confidence interval
D	diopter
DED	dry eye disease
IOL	intraocular lens
IOP	intraocular pressure
MGD	meibomian gland dysfunction
N	number
SD	standard deviation
SE	spherical equivalent
TBUT	tear break-up time
UDVA	uncorrected distance visual acuity

## References

[1] Lee C.M., Afshari N.A. (2017). The global state of cataract blindness. Curr. Opin. Ophthalmol..

[2] Lapp T., Wacker K., Heinz C., Maier P., Eberwein P., Reinhard T. (2023). Cataract surgery-indications, techniques, and intraocular lens selection. Dtsch. Arztebl. Int..

[3] Haddad N.M., Sun J.K., Abujaber S., Schlossman D.K., Silva P.S. (2014). Cataract surgery and its complications in diabetic patients. Semin. Ophthalmol..

[4] Takabatake R., Takahashi M. (2021). Preoperative factors affecting visual acuity following the implantation of diffractive multifocal intraocular lenses. J. Refract. Surg..

[5] Shukhaev S.V., Pustozerov E., Boiko E.V., Kirillova O.V. (2024). The accuracy of the trifocal iol calculation using equivalent k-readings and total corneal power in different zones. Graefes. Arch. Clin. Exp. Ophthalmol..

[6] Levin H.J., Mehta M.S., Storey P.P., Patel S.N., Kuley B., Wibbelsman T.D., Obeid A., Garg S., Vander J., Dunn J.P. (2023). Endophthalmitis following cataract surgery: Visual outcomes, microbial spectrum and complications. Curr. Opin. Ophthalmol..

[7] Chiu C.S. (2014). 2013 update on the management of posterior capsular rupture during cataract surgery. Curr. Opin. Ophthalmol..

[8] Li L., Wang X., Liu C., Wang S., Wang X. (2024). Incidence rate of secondary glaucoma following congenital cataract surgery: An in-depth systematic review and meta-analysis. Am. J. Ophthalmol..

[9] Briceno-Lopez C., Burguera-Giménez N., García-Domene M.C., Díez-Ajenjo M.A., Peris-Martínez C., Luque M.J. (2023). Corneal edema after cataract surgery. J. Clin. Med..

[10] Miura M., Inomata T., Nakamura M., Sung J., Nagino K., Midorikawa-Inomata A., Zhu J., Fujimoto K., Okumura Y., Fujio K. (2022). Prevalence and characteristics of dry eye disease after cataract surgery: A systematic review and meta-analysis. Ophthalmol. Ther..

[11] Donthineni P.R., Deshmukh R., Ramamurthy C., Sangwan V.S., Mehta J.S., Basu S. (2023). Management of cataract in dry eye disease: Preferred practice pattern guidelines. Indian J. Ophthalmol..

[12] Sheppard J., Shen Lee B., Periman L.M. (2023). Dry eye disease: Identification and therapeutic strategies for primary care clinicians and clinical specialists. Ann. Med..

[13] Nair S., Kaur M., Sharma N., Titiyal J.S. (2023). Refractive surgery and dry eye—An update. Indian J. Ophthalmol..

[14] Mencucci R., Vignapiano R., Rubino P., Favuzza E., Cantera E., Aragona P., Rolando M. (2021). Iatrogenic dry eye disease: Dealing with the conundrum of post-cataract discomfort. A P.I.C.A.S.S.O. Board narrative review. Ophthalmol. Ther..

[15] Messmer E.M. (2015). The pathophysiology, diagnosis, and treatment of dry eye disease. Dtsch. Arztebl. Int..

[16] Baudouin C., Messmer E.M., Aragona P., Geerling G., Akova Y.A., Benítez-del-Castillo J., Boboridis K.G., Merayo-Lloves J., Rolando M., Labetoulle M. (2016). Revisiting the vicious circle of dry eye disease: A focus on the pathophysiology of meibomian gland dysfunction. Br. J. Ophthalmol..

[17] Sweeney D.F., Millar T.J., Raju S.R. (2013). Tear film stability: A review. Exp. Eye Res..

[18] Seen S., Tong L. (2018). Dry eye disease and oxidative stress. Acta Ophthalmol..

[19] Uchino M., Yokoi N., Uchino Y., Dogru M., Kawashima M., Komuro A., Sonomura Y., Kato H., Kinoshita S., Schaumberg D.A. (2013). Prevalence of dry eye disease and its risk factors in visual display terminal users: The osaka study. Am. J. Ophthalmol..

[20] Fjaervoll H., Fjaervoll K., Magno M., Moschowits E., Vehof J., Dartt D.A., Utheim T.P. (2022). The association between visual display terminal use and dry eye: A review. Acta Ophthalmol..

[21] Barabino S. (2022). Is dry eye disease the same in young and old patients? A narrative review of the literature. BMC Ophthalmol..

[22] Mastropasqua L., Barboni P., Savini G., Aragona E., D'Aloisio R., Lanzini M., Agnifili L., Galzignato A., Solimeo A., Knutsson K.A. (2023). Refractive surgery and dry eye. Eur. J. Ophthalmol..

[23] Quinto G.G., Camacho W., Behrens A. (2008). Postrefractive surgery dry eye. Curr. Opin. Ophthalmol..

[24] Dossari S.K. (2024). Post-refractive surgery dry eye: A systematic review exploring pathophysiology, risk factors, and novel management strategies. Cureus.

[25] Dohlman T.H., Lai E.C., Ciralsky J.B. (2016). Dry eye disease after refractive surgery. Int. Ophthalmol. Clin..

[26] Toda I. (2018). Dry eye after lasik. Investig. Ophthalmol. Vis. Sci..

[27] Krance S.H., Hatamnejad A., Uddin R., Somani S., Tam E., Murtaza F., Chiu H.H. (2024). Health care utilization, prevalence, and risk factors of dry eyes after cataract surgery. Can. J. Ophthalmol..

[28] Garg P., Gupta A., Tandon N., Raj P. (2020). Dry eye disease after cataract surgery: Study of its determinants and risk factors. Turk. J. Ophthalmol..

[29] Sahu P.K., Das G.K., Malik A., Biakthangi L. (2015). Dry eye following phacoemulsification surgery and its relation to associated intraoperative risk factors. Middle East Afr. J. Ophthalmol.

[30] Lin B., Li D.K., Zhang L., Chen L.L., Gao Y.Y. (2024). Postoperative dry eye following femtosecond laser-assisted cataract surgery: Insights and preventive strategies. Front. Med. (Lausanne).

[31] Al-Osaily A.M., Al-Jindan M.Y. (2018). Intra-correlations between cataract density based on scheimpflug image, phacodynamics, surgery duration, and endothelial cell loss after phacoemulsification. Saudi J. Ophthalmol..

[32] Szczotka-Flynn L.B., Maguire M.G., Ying G.S., Lin M.C., Bunya V.Y., Dana R., Asbell P.A. (2019). Impact of dry eye on visual acuity and contrast sensitivity: Dry eye assessment and management study. Optom. Vis. Sci..

[33] Bron A.J., Evans V.E., Smith J.A. (2003). Grading of corneal and conjunctival staining in the context of other dry eye tests. Cornea.

[34] Qu H., Abulimiti A., Liang J., Zhou S., Wu Z., Chen Y., Ju R., Wang Z., Xu R., Chen X. (2024). Comparison of short-term clinical outcomes of a diffractive trifocal intraocular lens with phacoemulsification and femtosecond laser assisted cataract surgery. BMC Ophthalmol..

[35] Wang X., Liu S., Chen Y., Gong J., Wu N., Yao Y. (2024). Extended depth of focus iol in eyes with different axial myopia and targeted refraction. BMC Ophthalmol..

[36] Agard E., Levron A., Billant J., Douma I., Dot C. (2024). Comparison of refractive outcomes obtained with two swept-source oct-based optical biometers after cataract surgery: A study of 152 eyes. J. Fr. Ophtalmol..

[37] Cao X., Shao J., Zhang Y., Zheng L., Zhang J. (2024). Long term evaluation of surgically induced astigmatism and corneal higher-order aberrations after 2.2 mm clear corneal incisions in femtosecond laser-assisted cataract surgery: Temporal versus superior approach. Clin. Ophthalmol..

